# Animal Models of Psychiatric Disorders That Reflect Human Copy Number Variation

**DOI:** 10.1155/2012/589524

**Published:** 2012-07-30

**Authors:** Jun Nomura, Toru Takumi

**Affiliations:** ^1^Laboratory of Integrative Bioscience, Graduate School of Biomedical Sciences, Hiroshima University, 1-2-3 Kasumi, Minami, Hiroshima 734-8553, Japan; ^2^Japan Science and Technology Agency (JST), CREST, Chiyoda, Tokyo 102-0075, Japan

## Abstract

The development of genetic technologies has led to the identification of several copy number variations (CNVs) in the human genome. Genome rearrangements affect dosage-sensitive gene expression in normal brain development. There is strong evidence associating human psychiatric disorders, especially autism spectrum disorders (ASDs) and schizophrenia to genetic risk factors and accumulated CNV risk loci. Deletions in 1q21, 3q29, 15q13, 17p12, and 22q11, as well as duplications in 16p11, 16p13, and 15q11-13 have been reported as recurrent CNVs in ASD and/or schizophrenia. Chromosome engineering can be a useful technology to reflect human diseases in animal models, especially CNV-based psychiatric disorders. This system, based on the Cre/*loxP* strategy, uses large chromosome rearrangement such as deletion, duplication, inversion, and translocation. Although it is hard to reflect human pathophysiology in animal models, some aspects of molecular pathways, brain anatomy, cognitive, and behavioral phenotypes can be addressed. Some groups have created animal models of psychiatric disorders, ASD, and schizophrenia, which are based on human CNV. These mouse models display some brain anatomical and behavioral abnormalities, providing insight into human neuropsychiatric disorders that will contribute to novel drug screening for these devastating disorders.

## 1. Introduction

Copy number variation (CNV) is a structural genomic variation of the human genome that may either be inherited or caused by de novo mutation. It includes translocation, inversion, duplication, triplication, and deletion. CNVs can range in size from kilobases (Kbs) to several megabases (Mbs) that have not been identified by conventional chromosomal analysis. However, recent technology of genome-wide analysis such as comparative genomic hybridization (CGH) has led to the discovery of extensive genomic structural variation [1–3]. A recent report using microarray technology revealed that as much as 12% of the human genome are variable in copy number [4]. These known CNVs are available from the interactive web-based database DECIPHER (Database of Chromosomal Imbalance and Phenotype in Humans Using Ensembl Resources, http://decipher.sanger.ac.uk/). The DECIPHER database is a Consortium comprised of an international network of more than 100 centers and has uploaded more than 2000 cases (current statistics can be found on the DECIPHER homepage) [5].

 CNVs can be de novo or familial. De novo mutations are more likely to contribute to the development of sporadic genomic disorders [6, 7]. In psychiatric disorders, ASD and schizophrenia, extension of genome-wide association studies (GWAS) have led to the discovery of both inherited and de novo sporadic CNVs. Such CNVs resulted in altering gene dosage and dosage-sensitive gene expression, which may contribute to these disorders complexities [8]. These human genetics studies have detected several CNVs (e.g., 1q21, 3q29, 10q26, 11p14, 15q11, 15q13, 16p13, 17p12, and 22q11). This discovery suggests an important role for the strict regulation of gene dosage in ASD and schizophrenia.

To understand psychiatric disorders, animal models are needed because particular experiments in human are impossible. While it is difficult to model human psychiatric phenotypes in animals (e.g., hallucinations and delusions characteristic of schizophrenia that are human specific), animal models may contribute to the elucidation of brain anatomy, behavioral characteristics, and molecular mechanisms that reflect aspects of human phenotypes. 

Although there is a strong association between genetic rearrangement and psychiatric disorders (e.g., ASD and schizophrenia), valid animal models that reflect etiology are rare. Several efforts have been made to generate mouse models of psychiatric disorders by conventional gene targeting, conditional gene targeting, and point mutation by chemical mutagens. But these techniques are not enough to reflect complex human genomic rearrangements, such as large deletions, inversions, and duplications. In this regard, the Cre/*loxP*-based chromosome engineering technique is useful to generate this kind of complex genomic rearrangements in the mouse genome. By using this chromosome engineering technique, we can accomplish CNV-based unbiased animal models of psychiatric disorders. In this paper, we focus on animal model of ASD (and schizophrenia) which was generated by chromosome engineering, principle of this technology, and discuss for future directions.

## 2. Chromosome Engineering in Mice

Genetic abnormalities such as point mutations, deletions, duplications, inversions, and translocations can be induced by exposure to X-ray radiation, chemical mutagens (e.g., N-ethyl-N-nitrosourea (ENU)), conventional gene-targeting, or chromosome engineering. X-ray causes DNA double-strand breaks, inducing genomic instability [16]. The chemical mutagen, ENU, induces single-base-pair substitutions in the genome causing mutations with partial functions [17, 18]. Animal models containing genes with point mutations can be used to reveal the gene's functional domain *in vivo* [19]. Both techniques are valuable but cannot predict the mutated position within the gene. Conventional gene targeting (replacement) is used to disrupt a gene (inserting markers or reporters) to determine a gene's function. Conditional gene-targeting utilizing the Cre/*loxP *and Flp/*FRT* system allows spatial and temporal control of gene expression. It has been increasingly used for gene function analysis *in vivo*.

**Figure 1:**
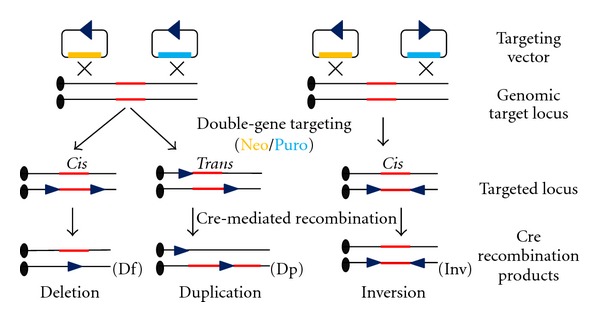
Chromosome engineering in mouse embryonic stem cells.

 Chromosome engineering is based on Cre/*loxP* technology, which can induce chromosome rearrangements (deletions, duplications [10, 20, 21], and inversions [22, 23]) in the mouse genome (Figure 1). 

Targeting vectors can be targeted in two orientations that result in deletion, duplication, or inversion. Each targeting vector has a *loxP* site and drug selection marker, neomycin resistance (Neo), or puromycin-resistant gene (Puro). *Cis* and *Trans* indicate *loxP* sites.

Two *loxP* sites are sequentially inserted by each targeting vector into the mouse embryonic stem (ES) cell genome. Each targeting vector contains a selection marker, neomycin, or puromycin resistance gene. The vectors are manipulated by hypoxanthine phosphoribosyl transferase (HPRT) expression following Cre recombinase expression in ES cells. Transient expression of Cre recombinase induces rearrangement between *loxP* sites in the mouse genome. Clones which carry the desired chromosomal rearrangement are identified by various methods: drug selection by hypoxanthine-aminopterin-thymidine (HAT) media, genomic Southern blot analysis, fluorescent in situ hybridization (FISH), and CGH (comparative genomic hybridization) array. Although CGH array cannot identify structural chromosome aberrations such as balanced reciprocal translocations and inversions, this technique is a powerful tool to detect CNVs from genome. 

To inactivate a target gene or locus by chromosome engineering, a gene target vector must be chosen or created. The Mutagenic Insertion and Chromosome Engineering Resource (MICER) (http://www.sanger.ac.uk/resources/mouse/micer/) was developed by Dr. Allan Bradley's group, the Wellcome Trust Sanger Institute and is useful as a gene-targeting vectors resource [24]. These ready to use targeting vectors can be accessed through the Ensembl mouse genome browser (http://www.ensembl.org/index.html). It is important to note that these targeting vectors use an insertion vector system rather than a replacement vector system. Given the same length of homologous sequence insertion vectors have a ninefold higher targeting efficiency than replacement vectors [25].

## 3. Animal Models Based on Human CNVs

In spite of a strong association between ASD (and schizophrenia) and CNV, animal models of CNV that reflect human genomic rearrangement are few. These animal models were generated by chromosome engineering and have several psychotic phenotypes similar to those seen in patients with genomic rearrangement (Table 1). In this section we focus on 15q11-13, 16p11.2, and 22q11 locus, which are well-known copy number variant linked to ASD (or/and schizophrenia).

### 3.1. 15q11-13 Duplication Syndrome (ASD)

Human chromosome region, 15q11-13, is a complicated region that contains **γ*-aminobutyric acidreceptor A (GABAA receptor)* clusters and several imprinting genes[26]. Paternally expressing genes include *MKRN3*, *MAGEL2*, *NDN*, and *SNURF-SNRPN*. Maternally expressing genes include *UBE3A* and *ATP10A*. In addition to these genes, this locus includes *noncoding small nucleolar RNAs (snoRNAs)* that are located between SNURF-*SNRPN* and *UBE3A*, which are paternally expressed and brain specific [27, 28]. Deletion or duplication of this locus causes severe neurological phenotypes. Prader-Willi syndrome (PWS) and Angelman syndrome (AS) are affected by changes in the 15q11-13 locus. Most notably, deletion of *UBE3A* has been identified to lead to AS phenotypes. Major clinical features of PWS include low birth weight, short stature, small hands and feet, severe hypotonia, feeding difficulties, obesity associated with hyperphagia starting in early childhood, mild to moderate mental retardation, and learning and behavioral problems including obsessive-compulsive disorder and autism [29, 30]. AS patients exhibit developmental delay, gait ataxia, balance disorder, frequent laughter/smiling, easily excitable personality, hyperactivity, speech impairment, microcephaly, seizures, epilepsy, and abnormal EEG (electroencephalogram) [31]. Additionally, AS patients often exhibit socialization and communication deficits, which are diagnostic criteria for ASD [32, 33].

Duplication of the 15q11-13 locus was first reported as a partial trisomy of chromosome 15 [34], and then two individuals with autistic disorder were reported [35]. This locus has been known as the most frequent cytogenetic abnormality in ASD [36, 37]. Generally patients with 15q11-13 duplication show hypotonia, delay in motor skills and language development, epilepsy, and cognitive and learning problems. Recently, Michelson et al. reported a patient with severe intractable epilepsy who has familial partial trisomy 15q11-13 inherited from a mother who has schizophrenia [38]. Autistic phenotype associated with 15q11-13 duplication, usually believed that maternal origin, *UBE3A* is involved [39–46]. Although maternal locus supposed to critical, paternally inherited patients had also developmental delay [44, 46–49]. Clinical reports have been accumulating but no mechanism has been addressed.

**Figure 2:**
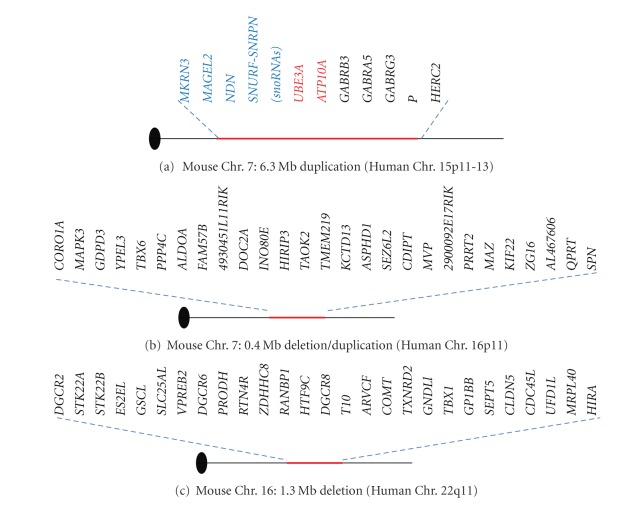
Schematic representation in studies applying chromosome engineering. Published in (a) Nakatani et al. [10], (b) Horev et al. [12], (c) Stark et al. [14]. The paternally, maternally expressed, and nonimprinting genes were labeled with blue, red, and black, respectively.

 To address this question, Nakatani et al. generated a mouse model of human 15q11-13 duplication [10]. This mouse was generated by chromosomal engineering based on the Cre/*loxP* system, and it has a 6.3 Mb duplicated locus in mouse chromosome 7c which is highly similar to human 15q11-13 (Figure 2(a)). 

Gene expression analysis revealed that paternally expressed genes, both Ndn and Snrpn, were twofold higher in paternally inherited mice (patDp/+) than wild-type (WT) mice. Similarly, maternally expressed gene Ube3a was twofold higher in maternally inherited mice (matDp/+) than WT mice. Histological analysis revealed no gross brain abnormalities. Monoamine levels in patDp/+ adult mice, serotonin (5-HT), and their metabolites 5-hydroxyindoleacetic acid (5-HIAA) were significantly downregulated in the midbrain and olfactory bulb. Also 5-HT content in developmental stage from postnatal 1 to 3 weeks in patDp/+ brain regions (cortex, hippocampus, cerebellum, midbrain, hypothalamus, pons, and medulla) was downregulated. This indicates 5-HT signaling during the developmental stage was significantly impaired in the brains of patDp/+ mice [10]. 5-HT influences not only mental condition (mood, social behavior, appetite, aggression and sleep) but also normal development of the central nervous system [50–52]. In addition to this, abnormal 5-HT levels have been found in ASD patient blood cells. For these reasons, 5-HT is one of the drug targets for ASD therapy. Treatment with serotonin reuptake inhibitors (SSRIs) have shown moderate success in recovering behaviors [53]. 

Behavioral tests revealed that patDp/+ mice display autistic behaviors such as less social interaction in the three-chamber social interaction test [54], abnormal ultrasonic vocalizations (USVs) [55] in postnatal developing pups separated from their dams, and behavioral inflexibility in the Morris Water Maze and Barnes Maze [10]. The phenotypes seen in patDp/+ mice indicate that these mice have impaired behaviors that include social interaction, communication, restricted interest, and resistance to change. These deficits correspond to human autistic phenotypes [56, 57]. Furthermore, patDp/+ mice showed anxiety-related phenotypes: decreased locomotor and exploratory activities in the open field and *Y*-maze test, and long latencies in novelty suppressed feeding test [11]. These anxiety-related phenotypes frequently accompany autistic symptoms in humans [58, 59]. Also the marble burying test, which is a useful test for the study of anxiety, obsessive-compulsive disorder (OCD), and neophobia, found that the number of buried marbles was significantly low in patDp/+ mice [11].

### 3.2. 16p11.2 Deletion/Duplication Syndrome (Deletion: ASD, Duplication: Schizophrenia)

Deletion or duplication of the chromosome 16p11.2 locus was observed in nearly 1% of multiplex families with ASD [60]. Meta-analysis of patients with ASD and/or developmental delay estimated that 16p11.2 locus deletion is associated with a 38.7-fold increase in the odds of ASD/developmental delay. On the other hand, 16p11.2 locus duplication is associated with a 20.7-fold increase in the odds of ASD/developmental delay [60–63]. In addition to these, 16p11.2 deletion is associated with obesity [64], and duplication is associated with schizophrenia [65] as well as ASD [60, 66]. Notably, a brain anatomical abnormality (abnormal head size) has been reported to be associated with this locus. For instance, patients with the 16p11.2 deletion had statistically significant macrocephaly and those with duplication had microcephaly [67].

 A mouse model of human 16p11.2 deletion (df/+) as well as duplication (dp/+) has been reported [12] (Figure 2(b)). This mouse model was generated by Cre/*loxP*-based chromosome engineering. This locus includes 27 genes, *SPN, QPRT, c16orf54, KIF22, MAZ, PRRT2, c16orf53, MVP, CDIPT, SEZ6L2, ASPHD1, KCTD13, LOC124446, HIRIP3, CCDC95, DOC2A, FAM57B, ALDOA, PPP4C, YPEL3, GDPD3, MAPK3,* and *CORO1A*.

 Young df/+ mice (before weaning) tend to be smaller than WT siblings, but as adults they are almost the same size as WT siblings and look healthy. Interestingly, 16p11.2 CNV mice, df/+ and dp/+ mice have opposite phenotypes. In a novel environmental cage, df/+ mice displayed longer distance traveled and time spent walking as compared with WT mice. In contrast, dp/+ mice traveled a shorter distance and spent less time walking as compared with WT mice. Additionally, df/+ mice were significantly active in both dark and light period. These results indicate that 16p11.2 locus affects not only physical activity but also diurnal activity and sleeping related symptoms. Also, a brain anatomical study using Magnetic resonance imaging (MRI) identified several regional changes in 16p11.2 CNV mice. For instance, df/+ mice showed increased volume of several brain regions (percentage of total brain volume): forebrain, superior colliculus, fornix, hypothalamus, mammillothalamic tract, medial septum, midbrain, and periaqueductal grey. These brain regional volumetric changes were more significant between df/+ and dp/+ than betweendf/+ and WT mice. 

### 3.3. 22q11.2 Deletion Syndrome (22q11.2DS, DiGeorge Syndrome (DGS), Velo-Cardio-Facial Syndrome (VCFS)) (Schizophrenia)

Microdeletion of chromosome 22q11 is found in 1 out of every 4000 live births, making it one of the most common interstitial deletions [68]. This 22q11.2 microdeletion causes craniofacial, cardiovascular abnormalities, immunodeficiency, hypocalcaemia, short stature, and cognitive dysfunctions [69–71]. Microdeletion of this region accounts for 1-2% of the cases of people with schizophrenia [72, 73]. Also, this locus accounts for up to 1-2% of cases of sporadic schizophrenia [74–76]. Some neuroanatomical changes have been reported in patients with 22q11.2DS as well. Volumetric reduction in total brain volume includes cortical regions (e.g., frontal, parietal, temporal, and occipital lobes), hippocampus, and cerebellum [77–86]. However, inconsistency in these neuroimaging reports may be due to the small numbers of subjects used and differences in methodology [72]. Yet these neuroanatomical reports are informative because some abnormalities are consistent with phenotypes of those who have non-22q11.2 DS-associated schizophrenia [76].

The majority of deletions in this locus are 3 Mb deletions (−90% of the cases), but 1.5 Mb deletions (<10% of the cases) contain 28 known genes which include critical genes and increased risk of mental disorders [73, 87]. 

 The mouse chromosome 16 region is conserved with human 22q11.2. Animal models of the human 22q11.2 deletion were generated by 2 groups, and both groups used chromosome engineering [14, 88] (Figure 2(c)). These mouse models, Df(16)A^+/−^ [14] and LgDel/+ [88], include 1.5 Mb critical regions, and both of them display several behavioral abnormalities, such as deficits in working memory, sensorimotor gating, and fear conditioning [14, 89–92]. Working memory deficits are becoming one of the main features of patients with schizophrenia, thus these animal models are supposed to reflect some aspects of 22q11.2 DS syndrome phenotype. In addition to behavioral abnormalities in this mouse, diminished 22q11 locus dosage disrupts cortical neurogenesis, interneuron migration [93], dendritic complexity, and formation of excitatory synapses [94]. Although, several interesting phenotypes have been reported in this mutant mouse, there are no studies published about brain structural abnormalities even though several brain abnormalities have reported in human studies. These brain anatomical changes and molecular mechanisms that underlie these phenotypes will be interesting to elucidate and will be addressed by using brain imaging techniques.

## 4. Future Perspectives

Application of new technologies, such as Comparative Genomic Hybridization (CGH) and next-generation sequencing, will reveal more additional genomic rearrangements related to psychiatric disorders. Thus, to analyze both phenotypes and underlying molecular mechanisms that originate from genetic rearrangements, animal models will be a powerful tool. In this context, chromosome engineering will be a valuable tool. Recently Ruf et al. [95] reported that they generated several hundred mice and embryos which have one *loxP* and LacZ site at a random genomic positions that inserted by sleeping beauty-based transposition system. These lines are mapped in Transposon and Recombinase Associated Chromosomal Engineering Resource database (TRACER, http://tracerdatabase.embl.de/fmi/iwp/res/iwp_home.html). This database is useful in creating chromosome rearrangements *in vivo*.

A logical next step is to identify responsible gene(s) in CNV. It is an orthodox approach to narrow down the region by systematically insertion of *loxP* combining the existed lines such as above TRACER. Generating Bacterial Artificial Chromosome (BAC) transgenic mice is another way to identify critical genes. BAC transgenes inserted to the genome faithfully recapitulate chromosomal endogenous gene expression, since BAC transgenic mice may appropriate animal model of gene duplication. Also transient overexpressing (or knockdown) each transcript in developmental brain is possible strategy. Recently Golzio et al. [96] identified a responsible gene *KCTD13* in 16p11.2 locus which causes brain malformation by using zebrafish. Use of these technologies in generating valid and etiology-based animal model of psychiatric disorders will contribute to the development of drugs against disorders and elucidation of molecular mechanisms that underlie these psychiatric disorders.

## Figures and Tables

**Table 1:** Behavioral phenotypes of mouse models.

Human chromosomal region	Behavioral phenotypes	Reference
7q11.23 (deletion)	Increased sociability	[9]
	Increased acoustic startle response	
	Cognitive deficits	
	Growth retarded (male)	

15q11-13 (duplication)	Decreased sociability	[10, 11]
	Behavioral inflexibility	
	abnormal ultrasonic vocalizations	
	decreased spontaneous activity	
	Increased anxiety	

16p11.2 (deletion)	Hyperactive	[12]
	difficulty adapting to change	
	sleeping abnormalities	
	repetitive or restricted behaviors	

16p11.2 (duplication)	Hypoactive	[12]

17p11.2 (deletion)	cranio facial abnormalities	[13]
	Seizures	
	Obesity	

22q11.21 (deletion)	Deficits in sensorimotor gating	[14, 15]
	Working memory deficit	
	Deficit in both cued and contextual fear memory	
